# Contrastive Learning for Fault Detection and Diagnostics in the Context of Changing Operating Conditions and Novel Fault Types

**DOI:** 10.3390/s21103550

**Published:** 2021-05-20

**Authors:** Katharina Rombach, Gabriel Michau, Olga Fink

**Affiliations:** Swiss Federal Institute of Technology, ETH Zürich, 8092 Zürich, Switzerland; rombachk@ethz.ch (K.R.); gmichau@ethz.ch (G.M.)

**Keywords:** contrastive learning, triplet loss, fault diagnostics, fault detection

## Abstract

Reliable fault detection and diagnostics are crucial in order to ensure efficient operations in industrial assets. Data-driven solutions have shown great potential in various fields but pose many challenges in Prognostics and Health Management (PHM) applications: Changing external in-service factors and operating conditions cause variations in the condition monitoring (CM) data resulting in false alarms. Furthermore, novel types of faults can also cause variations in CM data. Since faults occur rarely in complex safety critical systems, a training dataset typically does not cover all possible fault types. To enable the detection of novel fault types, the models need to be sensitive to novel variations. Simultaneously, to decrease the false alarm rate, invariance to variations in CM data caused by changing operating conditions is required. We propose contrastive learning for the task of fault detection and diagnostics in the context of changing operating conditions and novel fault types. In particular, we evaluate how a feature representation trained by the triplet loss is suited to fault detection and diagnostics under the aforementioned conditions. We showcase that classification and clustering based on the learned feature representations are (1) invariant to changing operating conditions while also being (2) suited to the detection of novel fault types. Our evaluation is conducted on the bearing benchmark dataset provided by the Case Western Reserve University (CWRU).

## 1. Introduction

Modern industrial processes are increasingly subject to oversight by condition monitoring (CM) devices. The recorded data opens up the possibility of data-driven maintenance models [1]. Purely data-driven solutions are especially interesting with regard to complex assets for which model-based approaches are limited or do not exist. Recent successes in deep learning have demonstrated the potential of data-driven solutions [2,3]. However, for the task of fault detection and diagnostics, particular challenges arise when applying deep learning to CM data from an industrial asset.

Complex industrial assets are often subject to a variety of operating conditions as well as external (e.g., environmental) factors that strongly influence the acquired data. Changing ambient temperature, for example, might affect the roughness of the asset, which could then be sensed by accelerometer measurements resulting in changes of the signals. The ambient temperature is therefore a factor that causes variations in the data but cannot be controlled. This means that a complete training dataset that is recorded in summer will deviate from the data experienced in the winter season. Predicting or foreseeing all of these influential factors is not always possible as some factors of variations are simply not known or cannot be controlled. Even if all future operating conditions are completely controllable and known (e.g., defined in the specifications of a working environment), the multitude of possible combinations makes it often infeasible to collect a dataset with a sufficient representation of all possible combinations of operating conditions within the specifications. Hence, a training dataset might only represent a subset of all possible conditions. Ultimately, often in real applications, it is not realistic to assume that a training dataset contains all possible future conditions that the asset will experience [4]. In this paper, we distinguish between conditions or factors that are represented in the training dataset and those that are not. The later ones are referred to as **novel** operating conditions. However, the performance of data-driven models often relies on the fact that the data collected during inference time is similar to the training dataset (independent and identically distributed (IID)) [5]. For example, the training dataset needs to be representative of all ambient factors and operating conditions that the asset will encounter in the future. If a model is subjected to new variations in the data caused by, e.g., unexpected ranges of ambient temperature, it might perform poorly in identifying the exact system condition of the asset [6]. This can result in false alarms. To prevent this, a fault diagnostic model needs to be invariant to all variations in the data that correspond solely to varying operational or environmental factors rather than to a change in the asset’s condition.

On the other hand, while faults arise very rarely in operating industrial assets, there is a multitude of different fault types with various severities that can possibly occur [4]. It is not realistic to assume that the training dataset contains all possible fault types at all possible intensities. However, robust fault diagnostics entail the task of identifying fault types in general. This includes that those faults that are unknown at training time and, therefore, are not represented in the training dataset. Similarly to the terminology used for operating conditions that are not reflected in a training dataset, we refer to these faults as **novel** fault types. A safety issue can arise if a model is not capable of detecting novel fault types or is underestimating a fault’s severity. Therefore, to ensure safe operation, a robust fault diagnostic model needs to be sensitive to novel variations in the data that correspond to novel fault types.

Ultimately, the goal is to train a fault diagnostics model that is both invariant to the variability in the CM data caused by novel operating conditions or external factors and, simultaneously, sensitive to the changes corresponding to novel fault types that were not considered or known when the model was developed. In this work, we show that features trained with contrastive learning are able to achieve both of the aforementioned objectives. This is the first work that applies contrastive learning to PHM applications in order to tackle both of the above objectives: (1) invariance of the models to novel operating conditions and (2) sensitivity of the models with respect to variations caused by novel fault types.

## 2. Related Work

**Contrastive learning** is a discriminative approach that aims to group semantically similar samples close to each other in the feature space while pushing semantically dissimilar samples far apart from each other [7,8]. To achieve this, a contrastive loss is formulated based on a similarity metric quantifying how close different features are [9]. In contrast to other frequently used losses—such as cross-entropy loss or mean squared error loss, whose objective is to directly predict a label or values—contrastive learning aims to train a semantically meaningful feature representation of the data. This has recently shown great promise, mainly in the context of computer vision, and achieving or exceeding state-of-the-art results in both a supervised [8,9,10] and unsupervised setting [11,12,13]. Franceschi et al. applied contrastive learning also successfully to timeseries data [14].

If the contrastive loss function is based on triplets of training data samples, it is referred to as triplet loss. The idea of using data triplets (instead of data pairs) for contrastive learning was first introduced in 2009 for nearest-neighbor classification [15]. For each sample (the “anchor” $x_{a}$), the distance to both a positive sample ($x_{p}$) and a negative one ($x_{n}$) is calculated in order to formulate the loss function. Different techniques have been proposed to select these positive and negative samples. For supervised tasks, for example, the hard triplet loss [8] chooses the sample with the same label that is farthest away from the anchor ($x_{a}$) as the positive sample, whereas the nearest sample with a different label is selected as the negative sample. By contrast, the soft margin loss function [16] randomly selects a negative sample and regards all samples with the same labels within the batch as the positives. Regardless of the exact implementation, the objective is to group data with the same label and increase the distance to other classes of data in the feature space, i.e., to give the feature clusters a semantic meaning.

**Feature extraction or learning** has been identified as one of the most important elements in PHM applications [17]. Manually engineered features (feature extraction) as well as learned features (feature learning) have been proposed for the purpose of fault detection and diagnostics [18,19]. The resulting feature space is then classified [18,20] or clustered [19,21] in order to detect and classify faults and their severity, but also to detect novel fault types [19]. Robust feature learning is the objective of many publications of fault diagnosis [22,23]. These works typically focus on robustness with respect to noisy environments. This means that they assume to have representative (but noisy) samples of all classes. On the contrary, this paper focuses on robustness with respect to a shift of the underlying data distribution e.g., caused by changing operating conditions. Contrastive learning has been applied in domain adaptation settings for PHM applications (see below) [24] but not yet for robust feature learning in the context of unknown changing conditions and novel fault detection. However, the idea of learning low-dimensional representations of high-dimensional data that correspond solely to their semantic meaning is very promising. It offers the potential to filter out variations of the data that are caused by changing conditions and do not contain information regarding the asset’s condition.

**Transfer learning** in general relaxes the hypothesis that the training data must be IID with the test data [5]. By transferring knowledge that is learned in source tasks to a related target task, it aims to alleviate the issue of insufficient training data [5,25]. This has attracted a lot attention in machinery fault diagnostics, where, for example, changing operating conditions or external factors cause a shift in the CM data that is not reflected in the training dataset [26]. Means of domain adaption—a branch of transfer learning—have been widely used to address the challenge of adapting a model to new conditions [27,28,29,30]. The approach of Wang and Liu where contrastive learning is used for domain adaptation is noteworthy. However, these approaches require both (a) a clear identification of the target domain and (b) representative data for all classes from this target domain. Pioneering work by Wang et al. [27] has enabled the application of domain adaptation even if certain class data (e.g., certain faults) is missing in the target domain. Nevertheless, it still requires to identify and foresee the target domain, which is not always possible (e.g., if these new conditions are caused by external factors that are neither known nor controllable). Furthermore, representative data of all classes are required in the source domain. This is not given if the novel emerging fault types are those that have not been anticipated before.

## 3. Methodology

Contrastive learning is evaluated in the context of the PHM application of detecting, classifying, and determining the type and severity of bearing faults. Specifically, we evaluate whether fault detection and diagnostics based on the learned feature representation is, on the one hand, invariant to variations in the CM data caused by novel operating conditions and, on the other hand, sensitive to variations caused by novel fault types. To achieve that, the retrieved features are both classified and clustered. The feature representations are learned via the semi-hard implementation of the triplet loss $L_{Triplet}$ [16], where the negative loss is calculated based on one negative sample that is randomly sampled within a batch. The positive loss is computed based on the average distance of all positive samples within the batch to the anchor sample. The distance metric used for all case studies is the L2 Norm. The feature learning models are then applied to test datasets that contain novel operating conditions in Case Study 1 and in Case Study 2 the models are exposed to novel fault types.

To evaluate the suitability of the learned feature representation for detecting and classifying known fault types (but also for detecting novel fault types), the learned features are classified and clustered. A support vector machine (SVM) is trained for classification. The classification performance showcases whether the models are affected by a change in the operating conditions. For the identification of novel fault types, the feature space is clustered with two different clustering algorithms: *Ordering points to identify the clustering structure* (OPTICS) [31] as well as k-means [32], for which the silhouette score [33] is used to determine the number of clusters.

**Figure 1:**
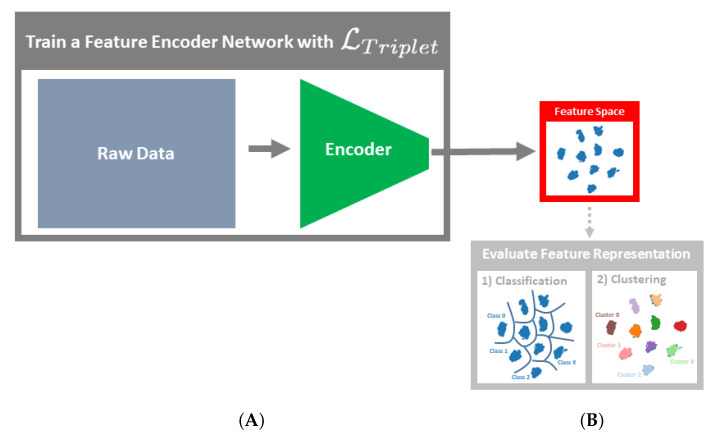
Methodology schemes of (**A**) training a feature representation with the triplet loss and (**B**) evaluating the learned feature representation (classification and clustering) with respect to the objectives of achieving invariance to novel operating conditions and sensitivity to novel faults.

A scheme of the methodology can be seen in Figure 1.

## 4. Case Studies

### 4.1. Dataset

All case studies are conducted on a bearing dataset provided by the Case Western Reserve University Bearing Data Center (CWRU dataset) [34]. The publicly available dataset is often used as a benchmark dataset in the field of PHM in general. It has been used for different tasks within the field of fault detection and diagnostics. Recently published methods include stacked denoising autoencoder [35] or recurrent neural networks [36] (a comprehensive overview is given by Neupane and Seok [37]). The dataset is especially suited to demonstrate solutions related to diagnosing faults under different operating conditions (different loads in this case) and transferring models between these different conditions (domain adaptation) [27,28,29,30].

However, we would like to emphasize that the setup that we are dealing with in this research has not yet been tackled by other researchers: the algorithms we are seeking to develop are on the one hand supposed to be sensitive to novel types of faults; however, on the other hand, they are supposed to be robust to novel operating conditions. Unfortunately, there are no other case studies that could be used to compare our proposed approach to directly. In fact, we reformulate the problem setup to make it applicable to the problem of novel fault type detection. Therefore, previous results obtained on this dataset are also not directly comparable.

The accelerometer measurements are recorded under four different loads 0, 1, 2, 3, which correspond to different operating conditions in our case studies. Ten different health conditions of the bearing are represented in the dataset (see Table 1): Healthy condition (N), three different fault types (inner race faults [IR], outer race faults [OR], and ball faults [B]), and three different fault severities for each of the fault types (7, 14, 21). The sample dataset was collected from the CWRU dataset with sampling frequency of 48 kHz.

**Preprocessing:** The original signals are divided into sequences of 512 points with no overlap between the sequences. Each sequence is scaled by the mean and standard deviation of the healthy data. This results in a dataset containing one-dimensional timeseries of length 512, each labeled by the label of the original signal.

The proposed algorithm and most of the baseline methods (see Section 4.3) uses raw signals as input data. However, we also compared the performance to that of algorithms based on feature engineering and used the frequently applied Fast Fourier Transform (FFT) for extracting features in the frequency domain [38].

The FFT features are calculated based on the previously extracted timeseries dataset whereby the absolute value of the FFT coefficients is considered as the FFT features. Due to the symmetry of the resulting features, only the first half is considered, resulting in a 256-dimensional feature space.

### 4.2. Case Study Setup

Two case studies are conducted to evaluate the suitability of contrastive learning with respect to the objectives of achieving (1) invariance of the models similar but novel operating conditions (interpolation—see Experiment (1)) as well as (2) sensitivity to novel fault types (extrapolation—Experiment (2)). In the following, these objectives and their corresponding setups are elaborated.

#### 4.2.1. Case Study 1: Invariance to Novel Operating Conditions

This case study tests the invariance of the trained models to novel changes in the operating conditions. As defined in the Section 1, novel operating conditions are those that are not represented in the training dataset. In the CWRU dataset, the different loads are considered as different operating conditions (see Section 4.1). The models are trained under a subset of operating conditions and evaluated on two test datasets: Data recorded under the same operating conditions as the training dataset (T) and a second test dataset containing data recorded under the operating condition that was not part of the training dataset ($T_{p}$). For example, if no data under the load 1 is available at training, the training dataset is defined as $D_{train}=$$D_{train}/D_{load=1}$ (19,129 samples) and the two test datasets are defined as 1) $T=D_{test}/D_{load=1}$ and 2) $T_{p}=D_{load=1}$. This case study setup corresponds to the scenario where a model experiences novel operating conditions or factors influencing the measurements during inference time that were not known at training time. The goal here is not to extrapolate to novel operating conditions but rather to train a feature representation that is not impacted by a shift in operating conditions. Therefore, the case study includes two data selections, whereby the two intermediate loads are being withheld for training. (This setup deviates from the typical experimental setup in the field of domain adaptation since we do not assume any knowledge about the missing conditions or target domain during training time.)

#### 4.2.2. Case Study 2: Sensitivity to Novel Fault Types

To test the ability of the model to distinguish known fault types and severities from novel ones, a model is trained on a subset of fault types and evaluated on two test datasets: One containing the same subset of fault types as the training dataset (T) and the second test dataset including the novel fault types that were not in the training dataset ($T_{p}$). The CWRU dataset used in this research (see Section 4.1) allows for multiple data selection choices to evaluate the objectives at hand. Two different exemplary data selections are chosen to evaluate the objective at hand—first, fault B is withheld from the training dataset and, second, the IR fault with all fault severities. For example, the first data selection results in a the training dataset $D_{train}=D_{train}/D_{fault=1,4,7}$ (18,195 samples) and the test datasets $T=D_{test}/D_{fault=1,4,7}$ (4549 samples) and $T_{p}=D_{fault=1,4,7}$ (4998 samples).

**Evaluation:** To evaluate the learned features with respect to the objective of achieving invariance to changing operating conditions, a classification model is trained based on the known classes at training time (see Section 4.4.2). To evaluate the objective of achieving sensitivity to novel fault types, the feature space of the test dataset containing the novel fault types is clustered. To evaluate the clustering performance, we closely follow the work of Arias Chao et al. [19] by reporting the following metrics: **R**: the number of detected clusters; AMI: the adjusted mutual information, measuring how closely the clustering algorithm replicates the true classes [39]; h: the homogeneity, which indicates whether clusters contain only data points which are members of a single class; c: the completeness, which measures whether members of a given class are elements of the same cluster [40]. Furthermore, a two-dimensional t-Distributed Stochastic Neighbor Embedding (t-SNE) [41] is used for visualization of the feature representation with a fixed perplexity value of 100.

### 4.3. Baseline Methods

Contrastive learning results in models that provide an informative feature representation of the data. To evaluate the performance of the contrastive learning framework, we defined several baseline models with the focus on encoding features in the latent space with different types of learning setups, ranging from supervised learning to autoencoding architectures. Different loss functions are used to optimize the encoder network. First, an autoencoder is trained with the objective to reconstruct the input signal with the mean squared error loss. The bottleneck layer activations provide the feature representation. Second, a classification model is directly trained to predict the labels with cross-entropy loss. The latent space activations provide the feature representation. To provide a clear comparison for the evaluation of the different loss functions with respect to the different objectives, the same encoder model architecture is used for all the encoding models (the concrete choice is explained in Section 4.4.1). Third, experiments are also conducted on features extracted from the raw input signals: Fast Fourier Transform (FFT) coefficients. The fourth evaluation model is an autoencoder architecture that is optimized with respect to the goal to ideally reconstruct the FFT coefficient and not the raw data.

### 4.4. Models

#### 4.4.1. Encoder

A small latent feature space dimensionality is chosen arbitrarily with the purpose of creating a bottleneck that needs to select the most informative content and, thus, may help to remove some factors of variability. Therefore, the dimensionality of the latent feature space was set to 16. The feature encoders share the same architecture—with one exception (see below). The architecture was chosen such that good performance could be achieved on all training objectives given a feature space of 16 dimensions. The encoder network consists of four 1D-convolution layers (64, 32, 16, 8 kernels) with a kernel size of 12, activated with Leaky ReLu (alpha = 0.5), followed by a MaxPooling (with strides of 2), and a Dropout layer (with a dropout rate of 0.1). The output of the convolution layers is flattened before passing it to a fully connected layer with 16 dimensions, again, activated by Leaky ReLu (alpha = 0.5). The triplet encoder has an additional L2 normalization layer. The classifier is followed by a fully connected layer with number of classes in the training dataset and softmax activation. The autoencoder (AE) model is followed by a decoder model (reverse architecture of the encoder). To enable convergence, all models are trained with the Adam optimizer for 100 epochs and a batch size of 64.

The process to encode the FFT features is elaborated in Section 4.1. While the fixed, small feature space size allows for comparison of the different feature spaces, training an autoencoder successfully (minimizing the reconstruction error of the input signal) required an adaption of the model architecture. Additionally, it is beneficial to train it on the FFT features and not on the raw signals (as often done in literature [37]). Therefore, a second autoencoder model to reconstruct the FFT features is trained to enable a fair comparison (see Section 4.3) with the following encoder architecture. It consists of four 1D-convolution layers (64, 32, 16, 8 kernels) with a kernel size of 12 and a stride of 2, activated with Leaky ReLu (alpha = 0.5). The output of the convolution layers is flattened before passing it to a fully connected layer with 64 dimensions, again, activated with Leaky ReLu (alpha = 0.5).

#### 4.4.2. Classification

To evaluate the performance of the learned or extracted features, a supervised architecture was chosen that uses the learned or extracted features as input. It is important to highlight that supervised evaluations are not feasible for all the case studies. For the supervised evaluation case studies, an SVM with a Radial Basis Function kernel is trained based on the learned or extracted feature representations. For the supervised classifier, the outputs of the classifier are used directly without training an additional SVM on the learned features as in the case of the other two models. In Section 4.5, the specific hyperparameters are shown.

#### 4.4.3. Clustering

Since particularly the discovery of novel fault types requires unsupervised evaluation of the feature space, clustering approaches were applied to the learned or extracted features. Two different clustering methods are used for comparison purposes: a partitioning clustering approach and a density-based clustering approach.

The features of the classifier encoder and the AE are scaled by the mean value before applying the clustering.

**OPTICS:** the density-based algorithm *Ordering points to identify the clustering structure* uses a distance metric to group points that are close to each other. Compared to density-based spatial clustering of applications with noise (DBSCAN) [42], OPTICS allows for clusters of varying density. The utilized implementation deviates from the original OPTICS algorithm by first performing k-nearest-neighborhood searches on all points. This is then used to calculate core distances in order to identify core sizes. For details, please refer to [43]. One benefit of using OPTICS is that it has the ability to detect “noisy samples” as outliers. These are samples that are not contained in any cluster as they are not density-reachable as defined in [31]. This property is particularly useful for detecting novel fault types.

**K-means + silhouette score:** K-means is a clustering algorithm which assigns each sample to the cluster with the nearest mean [44]. In our research, the number of clusters is determined by the silhouette score [33]. It measures how similar an object is to its own cluster (cohesion) as compared to other clusters (separation) based on the Euclidean distance.

### 4.5. Hyperparameter Tuning

The hyperparameters of the supervised classification algorithm SVM are tuned on a validation dataset split from the training dataset (see first columns in Table 2). Although the unsupervised clustering algorithms do not rely on the availability of labels, it is beneficial to tune certain hyperparameters. To do this, we again exploit the availability of the labeled training dataset: The minimum number of clusters considered for *Kmeans + Silhouette* was set to the number of classes in the training dataset (ten for case study 1 and seven for case study 2). The maximum number of clusters was set to a fixed value of 20. When applying *OPTICS*, the explicit clustering method can be chosen, as well as the minimal number of samples per class and the maximum distance between two samples ϵ for one to be considered as being in the neighborhood of the other. These parameters were tuned to achieve high performance on a fraction of the training dataset corresponding to the size of the dataset T. Whenever possible, the smallest fixed value of ϵ was chosen such that an AMI of 98% was achieved in the fraction of the training dataset. Otherwise, the value was set to infinity. Each setting is shown in Table 2.

## 5. Results

### 5.1. Case Study 1: Invariance to Novel Operating Conditions

**Figure 2:**
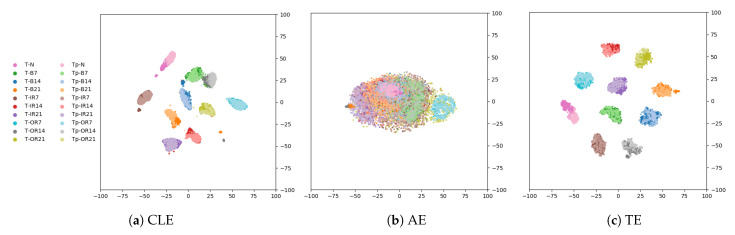
Case Study 1: t-SNE plot of feature space on $T\cup T_{p}$ of the classifier encoder (CLE), the Autoencoder (AE), and the Triplet Encoder (TE).

For visualization purposes, the 2D t-SNE of the feature spaces of the models that share the same encoder architecture (AE, the classifier encoder and the triplet encoder) are shown with the true labels ($y_{true}$) on $T\cup T_{p}$ in Figure 2. Exemplary, the figures of sample selection 1 ($D_{train}=$$D_{train}/D_{load=1}$) are displayed. Visually, the triplet encoder features appear to cluster the different classes best (see Figure 2). All clusters are well separated, cohesive, and contain only one class of data. The silhouette score per class confirms the visual impression (see Table 3), as the feature space of the triplet encoder shows the highest silhouette score calculated on the true labels. However, a slight deviation is visible within the classes from T to $T_{p}$.

Hence, the classification performance based on the triplet encoder features is not impacted by the change in operating conditions of the different test datasets (accuracy of 100% on T and $T_{p}$ on both of the sample selections—see classification results in Table 4). Similarly, the classification performance based on classifier encoder features is hardly impacted by the change of operating conditions—only a negligible performance drop of 1% is observed from T to $T_{p}$ for both sample selections—see Table 4. On the contrary, all other models show a more significant accuracy drop from test dataset T to $T_{p}$ on both sample selections (more pronounced in sample selection 1). This showcases the issue of changing operating condition for the fault diagnostic task and disqualifies these methods to be used in these scenarios of changing operating conditions.

Clustering methods are used for the second objective of detecting novel fault types (see Exp. 2). However, the clustering needs to perform well on $T\cup T_{p}$, even if no novel fault types—but rather only a shift in the operating conditions—are present. If this were not the case, it would not be possible to distinguish between variations in the data due to changes in the operating conditions and the presence of novel fault types. In Table 4, the clustering performance on the respective feature representations is shown. It is apparent that only the clustering of the triplet encoder feature space is not impacted by the change in operating conditions. Hardly any performance change is observed between clustering based on T and clustering based on $T\cup T_{p}$ on the sample selection 1 ($D_{train}=$$D_{train}/D_{load=1}$)—see clustering results in Table 4. For data selection 2 ($D_{train}=$$D_{train}/D_{load=2}$), a slight change is observed when using OPTICS (AMI changed by 3%). However, this is still the highest AMI compared to the other methods. Clustering based on the other features perform considerably worse. For example, on sample selection 1 ($D_{train}=$$D_{train}/D_{load=1}$), OPTICS underestimated the number of classes present in the feature spaces of the classifier encoder (R = 6) and the AE (R = 3). Hence, data from different classes are assigned to the same cluster, resulting in a lower *h* score compared to the *c* score. Clustering with k-means performs slightly better for all methods. This is due to the fact that the minimum number of clusters was set to 7 (see Section 4.5). Hence, the number of clusters is closer to the number of true classes in the data resulting in a better performance compared to the density-based clustering method OPTICS. We again see a higher *c* score compared to the *h* score for all evaluation models. This means that multiple classes are assigned to one cluster, whereas other classes are split into multiple clusters.

### 5.2. Case Study 2: Missing Faults

**Figure 3:**
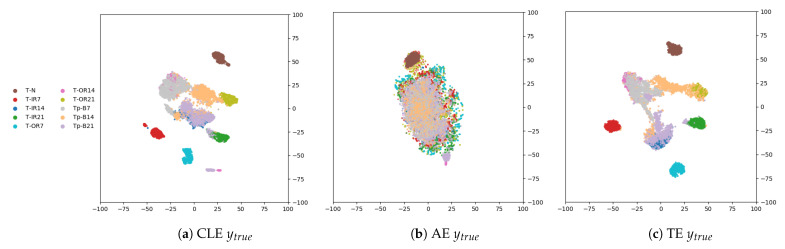

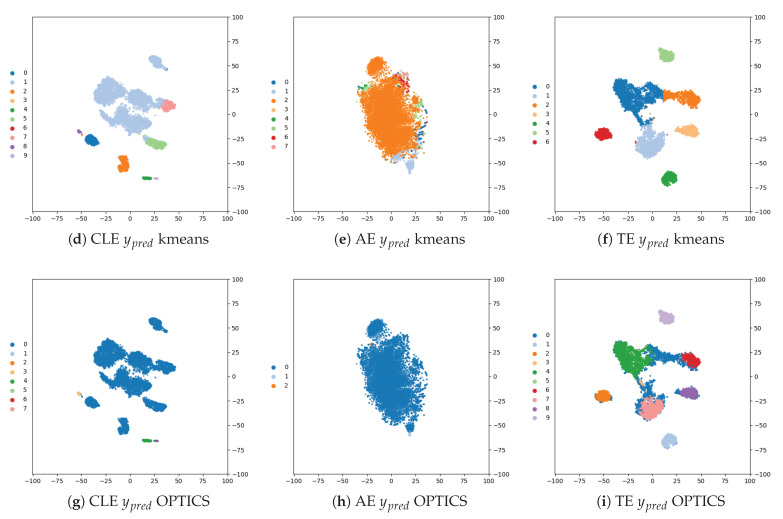
Case Study 2: t-SNE plot of feature space of the classifier encoder (first column), AE (second column), and triplet encoder (last column) model on $T\cup T_{p}$ with the true labels (first row), the predicted labels with k-means (second row), and the predicted labels with OPTICS (last row).

In Figure 3, the feature space is depicted for $T\cup T_{p}$ for sample selection 1 ($D_{train}=D_{train}/D_{fault=1,4,7}$) and the models sharing the same encoder architecture (AE, classifier encoder, triplet encoder). The true labels as well as the predicted cluster class of the two methods (OPTICS and k-means) is displayed.

In the first row of the figure, the true labels of the different feature spaces are shown. The light grey (fault B7), light orange (fault B14), and light purple (fault B21) correspond to the novel fault types. These are not well isolated in any of t-SNE visualizations, as can be seen in the first row Figure 3. Therefore, none of the clustering algorithms can identify the novel fault types as distinct clusters. However, the original clusters in T in the triplet encoder feature space are still being found in $T\cup T_{p}$ using both clustering methods (third column in Figure 3). While k-means simply assigns the novel fault types to the already existing clusters, OPTICS identifies some data of the novel fault types as outliers (labeled with 0): For example, on data selection 1 ($D_{train}=D_{train}/D_{fault=1,4,7}$), considering the triplet encoder feature space, a total of 2036 noisy samples or outliers are detected, of which 166 are B7 faults (11% of all B7 faults), 1140 are B14 faults (76% of all B14 faults), and 623 are B21 faults (43% of all B21 faults). Ultimately, 94% of the outliers are from the novel fault type.

The evaluation metrics are shown in Table 5. Only the class clusters of the triplet encoder are similarly compact such that a fixed value of ϵ could be set (see Section 4.5). Therefore, outliers could be identified as samples that are not density reachable. For the other baseline methods, this was not possible (see Section 4.5). As the novel faults are not well isolated in either of the resulting feature spaces, OPTICS performs well only in identifying novel faults as outliers on the triplet encoder features, where the cluster densities are compact. It is apparent that the feature space of the different AE as well as the FFT features does not provide a feature representation that is able to group the different fault types. This is also true for the feature space of the classifier. Many classes are grouped in the same cluster, whereas other classes are split into multiple clusters, resulting in a higher *c* score compared to the *h* score for both clustering methods.

## 6. Discussion

The goal of this research is to learn a feature representation that allows for robust classification under changing operating conditions as well as identification of novel faults. None of the goals are a classification task per se. However, the classification results on test dataset (T) allow for comparison with results of other State-of-the-Art (SOTA) publications on the used benchmark dataset. Accuracies above 99% have been achieved by various SOTA methods (see Section 4.1). Despite the rather simple model architectures evaluated in this paper (compared to other SOTA models—see Section 4.1), the classification results on the test dataset T of up to 100% showcase the validity of the proposed methods including the chosen baseline methods.

Over all the case studies, the performance based on the AE with the 16-dimensional feature space is very low. However, we consider a low-dimensional feature space more suited to filtering out uninformative variations from the input data, which is one of the objectives of this work. Therefore, we consider this a fair comparison. The lack of robustness of these autoencoding methods to new operating conditions becomes particularly apparent in the high classification performance drop in case study 1 from T to $T_{p}$ (see Table 4). This is not surprising as the AE is trained to fully reconstruct the input signal. Hence, the objective is to pass all information regarding the measurements through the bottleneck layer, including information related to various operating conditions. Therefore, variations in the operating conditions appear in the feature space as well, making this approach not suitable if the objective is to achieve invariance or robustness to operating conditions. Similarly, the FFT features contain all information of the signal including variations caused by operating conditions. Therefore, the classification performance based on these features are equally affected by the change in operating conditions.

The labels are directly considered when training the classifier encoder and triplet encoder, enabling the models to focus on the semantic meaning. This results in a better classification performance. Remarkably, the classification performance based on the classifier encoder and triplet encoder features is hardly affected if the operating conditions change at inference time. The clustering performance on the features of these two models varies significantly, both on T and $T\cup T_{p}$. As the features of a certain class are represented in a more compact way by the triplet encoder, the space is more suited for clustering. However, a shift can be visually observed between the data of T and $T_{p}$ within the respective clusters. This means that the model is not invariant to the shift in operating conditions. However, the different classes in $T\cup T_{p}$ are still cohesive and separable. Therefore, neither the classification nor the clustering performance is negatively impacted by the novel operating conditions. Both clustering methods perform well on $T\cup T_{p}$, with k-means even delivering results comparable to the classification performance.

All feature encodings are sensitive to variations in the data corresponding to novel faults. However, they do not provide a representation that allows the clustering algorithms to isolate them in the feature space. Therefore, none of the clustering methods identifies clusters including most of a novel fault class. However, the compactness of the learned feature representations per class of triplet encoder enables to set a fixed value of ϵ in OPTICS, i.e.,a fixed maximal value for two samples to be considered neighbors in a cluster. This enables us to detect novel faults at least as outliers (if not as distinct clusters). A detected outlier could raise an alarm to the operator and initiate a further evaluation. For example, in the sample selection 1 of case study 2, 94% of the outliers actually correspond to novel faults, and relatively few false alarms will be raised. However, many novel faults will not be detected but simply registered as another fault class. In this case, fault detection will still be ensured.

**Limitations:** The performance of the OPTICS clustering algorithm depends strongly on the data at hand: If the dataset contains mainly novel faults ($|T_{p}\left|>>|T\right|$), these will primarily determine the clusters and will not be detected as outliers anymore. Therefore, it is important to keep the dataset T with known conditions as a reference for the clustering algorithm. Continuously, a novel dataset with unknown conditions $T_{p}$ can be added. Our case studies have been conducted under an approximate balance between the two datasets ($\left|T|\approx \right|T_{p}|$); this ratio can be tuned according to the safety criticality of the system.

## 7. Conclusions

In this research, contrastive learning has been evaluated in the context of PHM applications. Specifically, two typical scenarios in PHM were investigated: a trained model is faced with new operating conditions and new faults at inference time. We were able to show that a feature representation trained with a contrastive learning paradigm is well suited to the clustering of classes under different and partially novel operating conditions. This enables clustering that is invariant to fluctuation in the data corresponding to similar but novel operating conditions, as seen before. Simultaneously, the compactness of the retrieved feature representations enables density-based clustering that is sensitive to novel faults. Ultimately, contrastive learning seems to be a promising paradigm for PHM applications. To further establish contrastive learning in PHM applications, we propose to dedicate future work to the question of how contrastive learning can be applied in a semi-supervised or unsupervised setting.

## Figures and Tables

**Table 1:** Classes in the CWRU dataset.

Class	0	1	2	3	4	5	6	7	8	9
Severity [mils]	-	7	7	7	14	14	14	21	21	21
Type	N	B	IR	OR	B	IR	OR	B	IR	OR

**Table 2:** Classification and clustering hyperparameters based on the feature spaces of the FFT, the Autoencoder based on the FFT (AE${}_{FFT}$), the Autoencoder (AE), the Classifier Encoder (CLE), and Triplet Encoder (TE) Models.

	Classification—SVM		Clustering—Exp. 1			Clustering—Exp. 2		
	C	γ	Method	#	ϵ	Method	#	ϵ
AE/AE ${}_{FFT}$/FFT	5.99	0.001	xi	10	∞	xi	10	∞
CLE	-	-	xi	10	∞	xi	10	∞
TE	1.67	0.046	DBSCAN	10	0.2	DBSCAN	10	0.08

**Table 3:** Silhoutte score of the class clusters in the feature representation based on the FFT features (FFT), the autoencoder with FFT features ($AE_{FFT}$), the autoencoder (AE), classifier encoder (CLE), and triplet encoder (TE) on $T\cup T_{p}$.

FFT	${\mathrm{AE}}_{\mathrm{FFT}}$	AE	CLE	TE
0.04	0.10	−0.18	0.38	0.81

**Table 4:** Case Study 1: Classification and clustering results on various operating conditions based on feature spaces of the FFT, the Autoencoder based on the FFT (AE${}_{FFT}$), the Autoencoder (AE), the Classifier Encoder (CLE), and Triplet Encoder (TE) models. (Bold indicates the best results).

	Classification		Clustering—OPTICS								Clustering—k-Means							
	T	$T_{p}$	T				$T\cup T_{p}$				T				$T\cup T_{p}$			
	acc	acc	R	AMI	h	c	R	AMI	h	c	R	AMI	h	c	R	AMI	h	c
Sample Selection 1: $D_{train}=$ $D_{train}/D_{load=1}$; $T=D_{test}/D_{load=1}$ and $T_{p}=D_{load=1}$																		
FFT	97%	91%	5	27%	16%	84%	5	26%	15%	89%	11	47%	41%	56%	10	53%	47%	60%
AE${}_{FFT}$	97%	91%	3	26%	17%	88%	6	26%	16%	87%	11	46%	40%	56%	10	46%	38%	58%
AE	67%	60%	3	1%	1%	36%	4	1%	1%	32%	11	29%	22%	46%	14	29%	22%	46%
CLE	**100%**	99%	6	23%	14%	67%	6	23%	13%	80%	11	70%	62%	81%	11	70%	61%	82%
TE	**100%**	**100%**	11	**96%**	**98%**	**95%**	11	**97%**	**98%**	**95%**	10	**100%**	**100%**	**100%**	10	**99%**	**99%**	**99%**
Sample Selection 2: $D_{train}=$ $D_{train}/D_{load=2}$; $T=D_{test}/D_{load=2}$ and $T_{p}=D_{load=2}$																		
FFT	97%	95%	6	26%	15%	87%	5	25%	15%	94%	11	47%	41%	56%	11	47%	39%	58%
AE${}_{FFT}$	97%	94%	7	6%	4%	42%	6	25%	15%	93%	10	46%	40%	56%	10	45%	38%	57%
AE	65%	59%	2	4%	2%	5%	3	2%	1%	4%	20	29%	23%	43%	20	30%	23%	45%
CLE	99%	98%	8	28%	17%	72%	7	26%	16%	80%	13	70%	63%	80%	11	70%	60%	85%
TE	**100%**	**100%**	11	**96%**	**97%**	**94%**	10	**93%**	**92%**	**95%**	10	**99%**	**99%**	**99%**	10	**99%**	**99%**	**99%**

**Table 5:** Case Study 2: Classification and clustering results with novel faults based on feature spaces of the FFT, the Autoencoder based on the FFT (AE${}_{FFT}$), the Autoencoder (AE), the Classifier Encoder (CLE), and Triplet Encoder (TE) Models. (Bold indicates the best results).

	Classification		Clustering—OPTICS								Clustering—k-Means							
	T	$T_{p}$	T				$T\cup T_{p}$				T				$T\cup T_{p}$			
	acc	acc	R	AMI	h	c	R	AMI	h	c	R	AMI	h	c	R	AMI	h	c
Sample Selection 1: $D_{train}=D_{train}/D_{fault=1,4,7}$; $T=D_{test}/D_{fault=1,4,7}$ and $T_{p}=D_{fault=1,4,7}$																		
FFT	**100%**	0%	4	37%	24%	86%	5	23%	13%	87%	9	57%	53%	61%	7	41%	31%	59%
AE${}_{FFT}$	**100%**	0%	4	37%	24%	86%	4	23%	13%	89%	8	51%	47%	57%	7	39%	30%	56%
AE	84%	0%	3	4%	2%	4%	3	3%	1%	43%	13	21%	17%	29%	8	16%	11%	35%
CLE	**100%**	0%	8	7%	4%	34%	8	7%	4%	41%	9	75 %	68 %	83%	5	54%	41%	80%
TE	**100%**	0%	8	**96%**	**98%**	**94%**	10	**73%**	**69%**	**77%**	7	**100%**	**100%**	**100%**	7	**72%**	**64%**	**82%**
Sample Selection 2: $D_{train}=D_{train}/D_{fault=2,5,8}$; $T=D_{test}/D_{fault=2,5,8}$ and $T_{p}=D_{fault=2,5,8}$																		
FFT	97%	0%	3	36%	23%	88%	6	23%	13%	85%	7	35%	28%	50%	7	50%	41%	65%
AE${}_{FFT}$	97%	0%	5	37%	24%	84%	5	23%	14%	87%	7	42%	36%	51%	11	53%	48%	59%
AE	73%	0%	2	1%	1%	3%	2	1%	0%	4%	18	26%	23%	36%	7	28%	21%	43%
CLE	**100%**	0%	9	36%	25%	68%	9	24%	14%	73%	7	66%	58%	77%	7	61%	50%	78%
TE	**100%**	0%	8	**97%**	**99%**	**96%**	7	**76%**	**65%**	**93%**	7	**99%**	**99%**	**99%**	7	**79%**	**71%**	**90%**
